# Supplementary figures and images for: A comprehensive overview of the Chloroflexota community in wastewater treatment plants worldwide
Source: mSystems. 2023 Nov 22;8(6):e00667-23. doi: 10.1128/msystems.00667-23 (PMC10746286; doi:10.1128/msystems.00667-23)

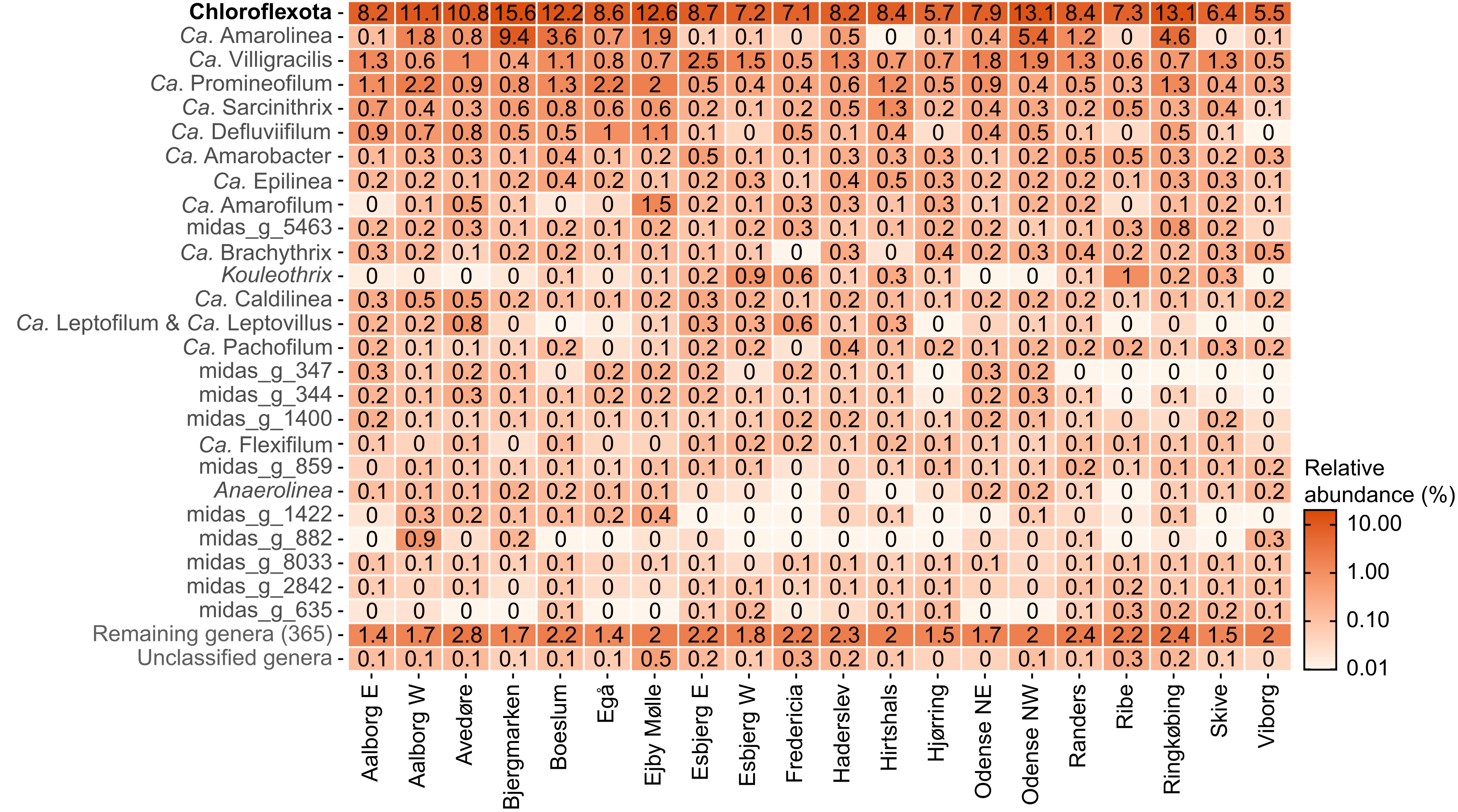

Supplement: Fig. S1 — Average mean abundance of the most abundant Chloroflexota genera in Danish WWTPs. Data were retrieved from the Danish MiDAS 3 survey (25). [file msystems.00667-23-s0003.png]

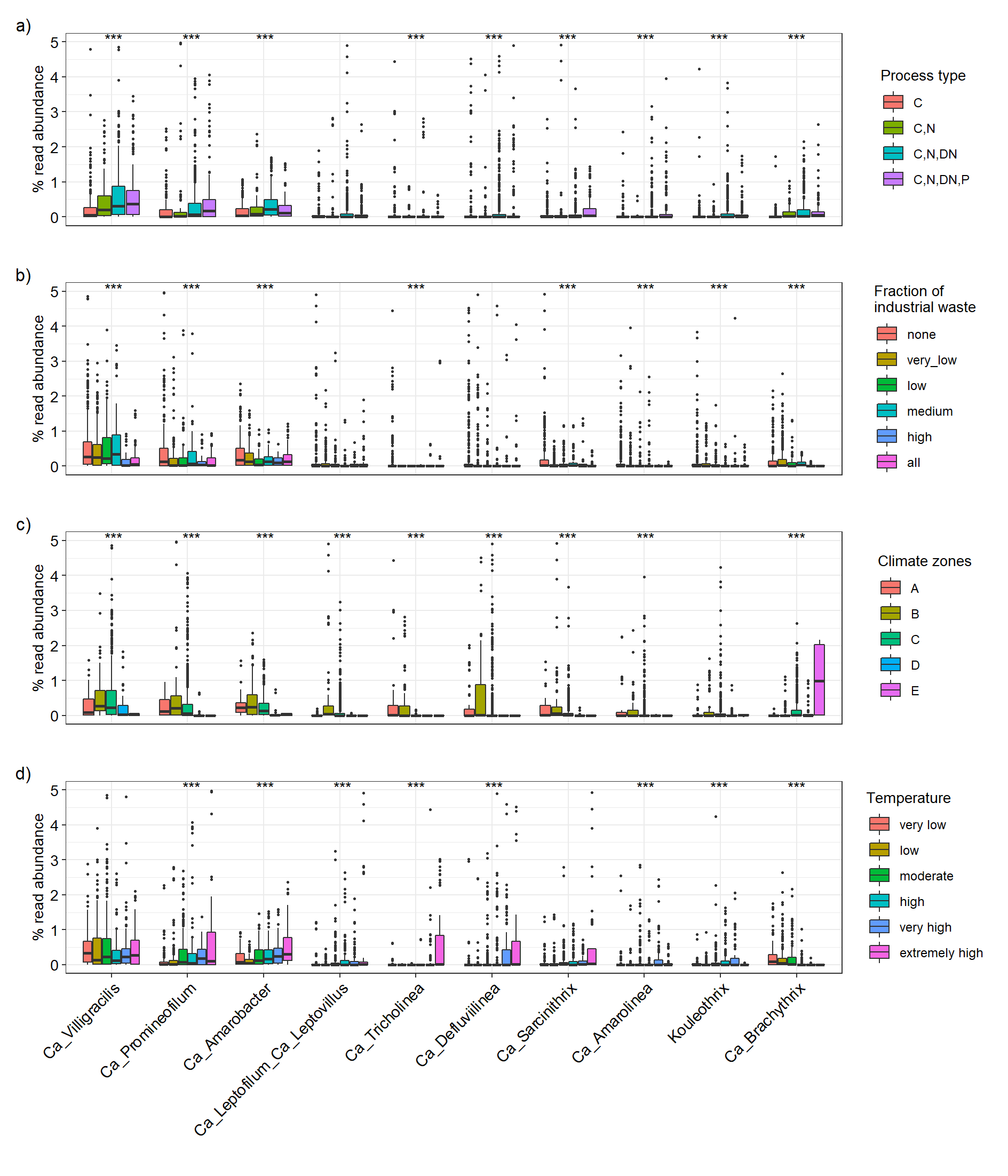

Supplement: Fig S2 — V1-V3 and V4 amplicon read abundance comparisons for the Chloroflexota phylum (first graph) and selected abundant genera. Data were retrieved from the global MiDAS survey (18) with a total of 929 activated sludge plants with different process designs. The gray diagonal line denotes equal V1-V3 and V4 abundances. For visualization purposes, samples with abundances higher than 10% are not shown in this figure. [file msystems.00667-23-s0004.png]

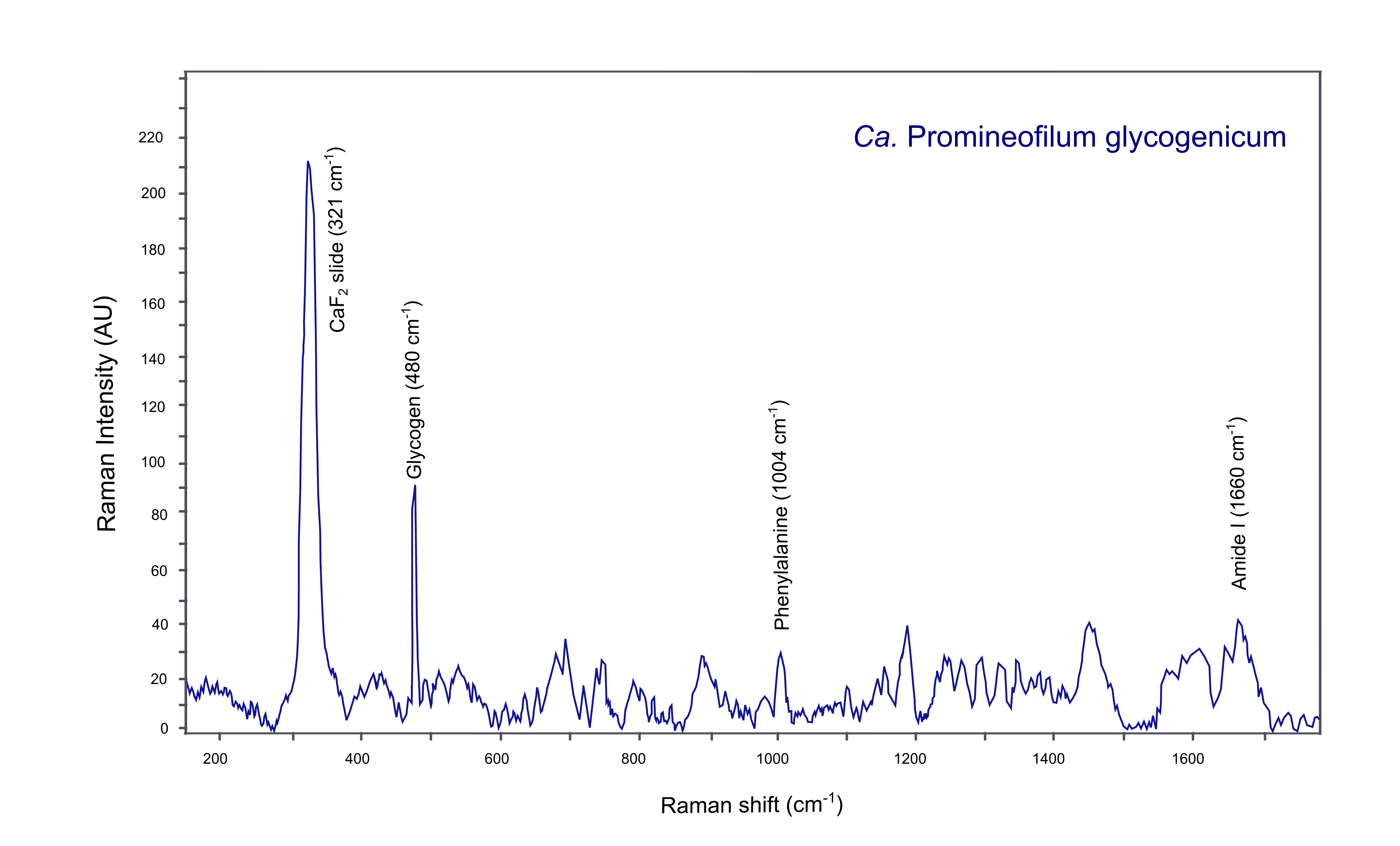

Supplement: Fig. S3 — Example of a Raman spectrum from the species Ca. Promineofilum glycogenicum, showing the presence of intracellular glycogen. [file msystems.00667-23-s0005.png]
