# Supplementary material for: A comprehensive overview of the Chloroflexota community in wastewater treatment plants worldwide
Source: mSystems. 2023 Nov 22;8(6):e00667-23. doi: 10.1128/msystems.00667-23 (PMC10746286; doi:10.1128/msystems.00667-23)
Supplement: Table S1 — Summary information about the climate zone division. [file msystems.00667-23-s0006.docx]

**Table S1**. Summary information about the climate zone division.

| **Abbreviation** | **Climate** | **Countries** |
| --- | --- | --- |
| **A** | Tropical/mesothermal | India, Malaysia, Philippines, Singapore |
| **B** | Dry (desert and semi-arid) | Argentina, Australia, Canada, China, Israel, India, Mexico, Saudi Arabia, Spain, USA |
| **C** | Temperate/mesothermal | Argentina, Australia, Austria, Belgium, China, Cyprus, Czech Republic, Denmark, Germany, Hong Kong, Israel, Italy, Netherlands, Norway, Portugal, Poland, Spain, South Africa, Switzerland, Sweden, UK, USA, Uruguay |
| **D** | Continental/microthermal | Canada, China, Finland, Norway, Poland, South Africa, Sweden, USA |
| **E** | Polar | Switzerland |
