# Supplementary material for: A comprehensive overview of the Chloroflexota community in wastewater treatment plants worldwide
Source: mSystems. 2023 Nov 22;8(6):e00667-23. doi: 10.1128/msystems.00667-23 (PMC10746286; doi:10.1128/msystems.00667-23)
Supplement: Table S2 — Detailed summary of the probes designed and optimized in this study. [file msystems.00667-23-s0007.docx]

**Table S2.** Detailed summary of the probes designed and optimized in this study.

| **Probe** | ***E. coli* pos.** | **Target group** | **Coverage**  **MiDAS4.8** | **Non-target hits** | **Sequence (5’-3’)** | **[FA]%** |
| --- | --- | --- | --- | --- | --- | --- |
| **CFX1111** | **1111-1132** | ***Ca.* Epilinea breve** | **17/20** | **0** | **CAC GTG AAA CAT ACG CCA AGG GT** | **40** |
| CFX1111_H1 | 1085-1106 | Helper for CFX1111 probe | N/A | N/A | GCG CTC GTT GCG GGA CTT AAC | N/A |
| CFX1111_H2 | 1135-1154 | Helper for CFX1111 probe | N/A | N/A | CGC CGG CAG TYG CGC ATG A | N/A |
| **CFX325** | **325-346** | ***Brachythrichaceae*** | **124/136** | **0** | **GTA GGC GTC TGG ACC GTG TTT** | **30** |
| CFX325_C1 | 325-346 | Competitor for CFX325 probe | N/A | N/A | GTA GGW GTC TGG ACC GTG TTT | N/A |
| CFX325_H1 | 299-320 | Helper for CFX325 probe | N/A | N/A | TCC TCT CAG AYC CCC TAC CCG | N/A |
| CFX325_H2 | 350-371 | Helper for CFX325 probe | N/A | N/A | TAT TCC TCM CTG CTG CCA CCC | N/A |
| **CFX198** | **198-219** | ***Ca.* Brachythrix** | **18/18** | **0** | **CCT CTC CTC ACG CCT TTC GAC** | **50** |
| CFX198_H1 | 170-194 | Helper for CFX198 probe | N/A | N/A | GAC CCT TTT GGG TAT TAG CCT CTC | N/A |
| CFX198_H2 | 230-249 | Helper for CFX198 probe | N/A | N/A | CTA GCT GAT GGG CCG CGG GCT | N/A |
| **CFX841_2** | **841-865** | ***Ca.* Trichofilum** | **33/33** | **0** | **AGC TAC AGC ACA GAG GGA TTG GAT** | **30** |
| CFX841_2_C1 | 841-865 | Competitor for CFX841_2 probe | N/A | N/A | AGC TAC AGC ACA GAG GGG TTG GAT | N/A |
| CFX841_2_C2 | 841-865 | Competitor for CFX841_2 probe | N/A | N/A | AGC TAC AGC ACA GAG GGA TTG GCT | N/A |
| CFX841_2_C3 | 841-865 | Competitor for CFX841_2 probe | N/A | N/A | AGC TAC AGC ACA GGG GGG TTG GAT | N/A |
| **CFX1086** | **1086-1110** | ***Flexifilaceae*** | **429/733** | **0** | **GCG CTC GTT TTC GGA CTT AAC CGA** | **30** |
| **CFX643** | **643-662** | ***Ca.* Flexifilum breve** | **116/119** | **0** | **TCC CAC TCT AGT CCC ACA G** | **30** |
| CFX643_C1 | 643-662 | Competitor for CFX643 probe | N/A | N/A | TCC CAC TCT AGT CCC GCA G | N/A |
| **CFX748** | **748-769** | ***Ca.* Leptofilum & *Ca.* Leptovillus** | **57/62** | **0** | **TTT CGC ATC TGA GCG TCA GGA** | **35** |
| CFX748_C1 | 748-769 | Competitor for CFX748 probe | N/A | N/A | TTT CGC ATC TGA GCG TCA GGT | N/A |
| CFX748_H1 | 720-738 | Helper for CFX748 probe | N/A | N/A | TGG CCC AGA GAG CCG CCT | N/A |
| CFX748_H2 | 740-760 | Helper for CFX748 probe | N/A | N/A | ATC CYG TTC TCT CCC CTA GC | N/A |
| **CFX1194** | **1194-1216** | ***Tepidiformales*** | **132/159** | **0** | **CGT AAG GGC CAC GCT GAC CTG A** | **50** |
| CFX1194_H1 | 1170-1190 | Helper for CFX1194 probe | N/A | N/A | TCG TCC CCT CCT TCC TCC GA | N/A |
| CFX1194_H2 | 1220-1241 | Helper for CFX1194 probe | N/A | N/A | GTA GCG TGT GTG TAG CCC CAG G | N/A |
| **CFX193** | **193-215** | ***Ca.* Amarobacter** | **33/104** | **0** | **TAG CGC CGG AGC TTT TAC CAC C** | **35** |
| CFX193_H1 | 168-192 | Helper for CFX193 probe | N/A | N/A | GGG TGT TAT GCG GTA TTA GCT CGC | N/A |
| CFX193_H2 | 220-240 | Helper for CFX193 probe | N/A | N/A | AGC TAA TCG GCC GCG GGC CC | N/A |
| **CFX122** | **122-141** | ***Ca.* Amarofilum** | **5/6** | **0** | **CTT GGG CAC ATT CCC ACG T** | **35** |
| CFX122_C1 | 122-141 | Competitor for CFX122 probe | N/A | N/A | TTT GGG CAC ATT CCC ACG C | N/A |
| **CFX682** | **682-706** | ***Ca.* Pachofilum** | **55/55** | **0** | **ATC TAC ATA TTC CAC CAT TAC ACC** | **35** |
| CFX682_H1 | 652-672 | Helper for CFX682 probe | N/A | N/A | TCC GCA TTC CTC TCA TYG CC | N/A |
| CFX682_H2 | 710-732 | Helper for CFX682 probe | N/A | N/A | CTT TCG CAC ATG AGC GTC AGG C | N/A |
| **CFX166** | **166-189** | ***Ca.* Tricholinea** | **14/17** | **0** | **GTA ACY TCA TGC GGT ATT AGC AG** | **35** |
| CFX166_H1 | 138-160 | Helper for CFX166 probe | N/A | N/A | GCA GGT CAC CAA CGC GTT ACT C | N/A |
| CFX166_H2 | 195-218 | Helper for CFX166 probe | N/A | N/A | GCT GAT GGG ACG CAG GCC CCT CC | N/A |
| **CFX1423** | **1423-1446** | ***Ca.* Defluviifilum** | **31/116** | **0** | **GAG TCA CCG ACT TCA GGT GTT CC** | **50** |
| CFX1423_C1 | 1423-1446 | Competitor for CFX1423 probe | N/A | N/A | AAG TCA CCG ACT TCA GGT GTT CC | N/A |
| CFX1423_C2 | 1423-1446 | Competitor for CFX1423 probe | N/A | N/A | AAG TCA CCG ACT TCA GGT GTT TC | N/A |
