# Supplementary material for: A comprehensive overview of the Chloroflexota community in wastewater treatment plants worldwide
Source: mSystems. 2023 Nov 22;8(6):e00667-23. doi: 10.1128/msystems.00667-23 (PMC10746286; doi:10.1128/msystems.00667-23)
Supplement: Table S3 — Abundance estimation (percentage of total) performed by 16S rRNA amplicon sequencing (V1-V3 regions primer set) and qFISH. [file msystems.00667-23-s0008.docx]

**Table S3.** Abundance estimation (percentage of total) performed by 16S rRNA amplicon sequencing (V1-V3 regions primer set) and qFISH.

| **WWTP** | **Sample date** | **Abundance (%)** | |
| --- | --- | --- | --- |
|  |  | **Sequencing** | **qFISH** |
| ***Ca.* Epilinea** | | | |
| Aars | August 2008 | 2.4 | 1.3 ± 0.3 |
| Boeslum | August 2007 | 1.4 | 1.6 ± 0.4 |
| ***Brachytrichaceae*** | | | |
| Oud-Turnhout (Belgium) | March 2018 | 2.2 | 6.6 ± 3.0 |
| Merelbeke (Belgium) | March 2018 | 2.1 | 2.2 ± 1.4 |
| ***Ca.* Brachythrix** | | | |
| Oud-Turnhout (Belgium) | March 2018 | 1.7 | 5 ± 1.2 |
| Merelbeke (Belgium) | March 2018 | 1.8 | 1.8 ± 0.5 |
| ***Ca.* Trichofilum**  Randers  Aars | August 2012  August 2008 | 0.6  0.5 | 0.8 ± 0.3  2.0 ± 0.8 |
| ***Flexifilaceae***  Fredericia  Kalundborg | August 2016  September 2017 | 2.6  2.8 | - 1. ±0.5   < 0.5 |
| ***Ca.* Flexifilum breve**  Esbjerg W  Hirtshals | August 2016  Hirtshals 2010 | 0.5  0.4 | < 0.5  < 0.5 |
| ***Ca.* Leptofilum & *Ca.* Leptovillus** | | | |
| Avedøre | August 2010 | 1.6 | 5.1 ± 1.5 |
| Aars | August 2007 | 0.8 | 1.6 ± 1.3 |
| ***Tepidiformales*** | | | |
| Randers | August 2010 | 1.2 | < 0.5 |
| Marselisborg | August 2006 | 1.0 | < 0.5 |
| ***Ca.* Amarobacter** | | | |
| Randers | August 2007 | 1.0 | < 0.5 |
| JeddahA (Saudi Arabia) | May 2018 | 3.0 | 1.1 ± 0.5 |
| ***Ca.* Amarofilum** | | | |
| Ejby Mølle | August 2008 | 8.3 | 3.8 ± 2.7 |
| Aars | August 2006 | 1.0 | 1.4 ± 1.3 |
| ***Ca.* Pachofilum** | | | |
| Aars | August 2009 | 2.8 | 2.4 ± 1.1 |
| Aars | August 2006 | 1.9 | 1.6 ± 0.8 |
| ***Ca.* Tricholinea** |  |  |  |
| Dahej (India) | July 2018 | 9.0 | 6.1 ± 1.6 |
| ***Ca.* Defluviifilum** |  |  |  |
| Åby | August 2007 | 1.5 | 9.9 ± 2.5 |
| Ejby Mølle | August 2018 | 1.2 | 5.2 ± 1.5 |
